# Pharmacogenetic testing in schizophrenia in real clinical practice: before or after antipsychotic -induced adverse drug reactions development?

**DOI:** 10.1192/j.eurpsy.2022.2066

**Published:** 2022-09-01

**Authors:** A. Abdyrakhmanova, N. Shnayder, R. Nasyrova

**Affiliations:** 1 FSBI “V.M. Bekhterev National Medical Research Centre of Psychiatry and Neurology” of the Health Ministry of the Russian Federation, The Centre Of Personalized Psychiatry And Neurology, St Petersburg, Russian Federation; 2 V.M. Bekhterev National Medical Research Centre of Psychiatry and Neurology, Centre Of Personalized Psychiatry And Neurology, Saint Petersburg, Russian Federation; 3 V.M. Bekhterev National Medical Research Centre for Psychiatry and Neurology, Centre Of Personalized Psychiatry And Neurology, Saint Petersburg, Russian Federation

**Keywords:** schizophrénia, pharmacogenetic testing, Adverse reactions, therapeutic resistance

## Abstract

**Introduction:**

Schizophrenia is socially significant mental disorder characterized by early onset and high time and financial expenditure on treatment. Antipsychotics (APs) are highly effective against positive and negative symptoms, but at same time have a wide range of adverse drug reactions (ADRs). APs efficiency and safety are variable and depend on characteristics of genetically determined mechanisms (transportation, biotransformation, and elimination).

**Objectives:**

Investigation role of pharmacogenetic testing (PhGT) on example of clinical case of severe ADRs in 47-year-old woman with schizophrenia.

**Methods:**

Patient’s medical history analysis; clinical observation; biochemical serum analysis; therapeutic drug monitoring; PhGT.

**Results:**

The clinical case of a woman with schizophrenia who has been noted to be unresponsive to APs for some years after schizophrenia onset. She was found to be homozygous for nonfunctional SNVs CYP2D6*4 and CYP2C9*2, heterozygous for CYP1A1*2A, which was reason for complete shutdown of isoenzymes 2D6, 2C9 and 1A1 activity and development of ADRs in use of initial doses of several APs, as well as for an increase in severity of ADRs with schizophrenia positive symptoms aggravation with an even slower titration of APs daily dose not only with polytherapy, but also with monotherapy. So, not recommented APs for patient: aripiprazole, haloperidol, zuclopenthixol, cariprazine, quetiapine, paliperidone, risperidone, thioridazine, sertindole, asenapine, alimemazine, chlorpromazine, etc. (CYP2D6); haloperidol, clozapine, olanzapine, perphenazine, promazine (CYP2C9); carefully: haloperidol, olanzapine, perospirone (СYP1A1).

**Conclusions:**

This rare case demonstrates PhGT importance before APs therapy, because the patient had very high risk AP – induced ADRs. She needed PhGt before APs use, but not after severe ADRs during 12 years.

**Disclosure:**

No significant relationships.

